# Construction and Application of Directed Acyclic Graphs in Leading Medical Journals

**DOI:** 10.1001/jamanetworkopen.2025.53803

**Published:** 2026-01-14

**Authors:** Guanghui Deng, Jian Du

**Affiliations:** 1 Institute of Medical Technology, Peking University, Beijing, China; 2 National Institute of Health Data Science, Peking University, Beijing, China

## Abstract

This cross-sectional study evaluates how directed acyclic graphs are constructed, reported, and applied for statistical adjustment across leading clinical journals.

## Introduction

Causal directed acyclic graphs (DAGs) constructed from literature and expert knowledge—rather than data-driven alone—are increasingly recommended to depict causal assumptions and prevent inappropriate adjustment in observational health research.^1,2,3,4^ However, how DAGs are actually constructed, reported, and applied for statistical adjustment across leading clinical journals remains unclear.

## Methods

For this cross-sectional study, we searched 4 major medical journal websites (*New England Journal of Medicine *[*NEJM*], *The Lancet*, *JAMA*, and *The BMJ*) and 4 related databases using a predefined set of terms for directed acyclic graphs. The complete search strategy is available in the eMethods in Supplement 1. The search identified 165 studies (10 in *NEJM*, 27 in *The Lancet*, 33 in *JAMA*, and 95 in *The BMJ*). We excluded nonoriginal or secondary studies, studies not applying DAGs, studies with unavailable DAGs, and studies where DAGs were not used for variable selection (Figure).

**Figure.**
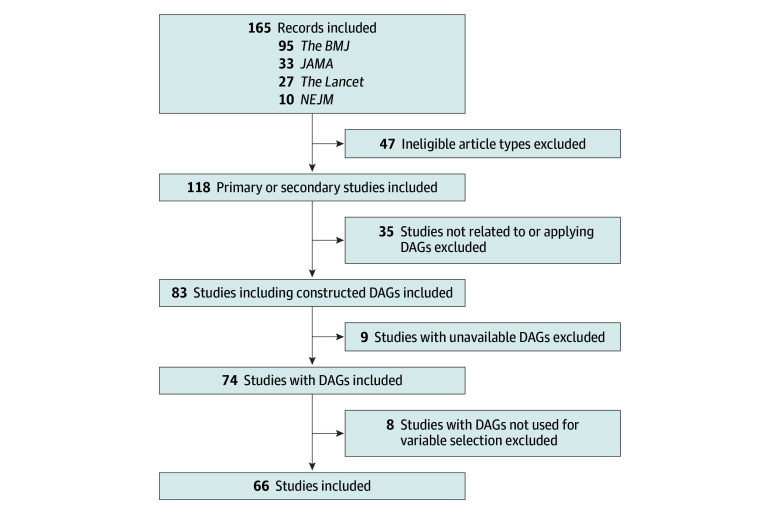
Study Flow Diagram DAGs indicates directed acyclic graphs; *NEJM, New England Journal of Medicine*.

For each DAG, we extracted (1) the study setting in which it was applied; (2) construction details, including whether the DAG was developed at the stage of study design and whether its edges were supported by literature and/or expert knowledge; and (3) applications, especially whether the DAG completely depicted confounders, mediators, and colliders and whether adjustment strategies implied by the DAG were consistent with those implemented in the analyses. All searches, screening, and data extraction were conducted independently by 2 reviewers (G.D. and J.D.). This review was conducted and reported in accordance with the Strengthening the Reporting of Observational Studies in Epidemiology (STROBE) reporting guideline. This study did not involve human participants directly and was therefore exempt from institutional review board approval under the Common Rule.

## Results

The characteristics of the included DAGs are presented in the Table. Among 66 studies containing 72 eligible DAGs, 65 (90%) were used in observational studies (60 cohort, 3 case-control, and 2 cross-sectional studies). Two DAGs (3%) were applied in interventional trials (1 nonrandomized and 1 randomized trial), and 5 DAGs (7%) were included in meta-analyses (4 individual participant data meta-analyses and 1 study-level meta-analysis). Only 26 of 72 (36%) explicitly stated that the DAG had been developed at the design stage. For edge justification, 11 of 72 DAGs (15%) cited published evidence; 11 of 72 (15%) stated there was supporting evidence without citation; and 50 of 72 (70%) did not specify. Expert knowledge involvement was reported in 11 of 72 DAGs (15%). Overall, 54% (39 of 72) of the studies exhibited inconsistencies between the covariate adjustment strategies suggested by their DAGs and those actually implemented in the analyses. Specifically, among 67 DAGs explicitly used for confounder identification, 19 (28%) failed to fully adjust for all specified confounders, largely due to missing data (15 of 19 DAGs [79%]); the remaining 4 cases were justified by author explanations. Conversely, 23 of 72 studies (32%) adjusted for variables not indicated by the DAG. These extra-DAG variables were predominantly nonconfounders (16 of 23 studies [70%]) or variables entirely absent from the original DAG (7 of 23 studies [30%]).

**Table.** Characteristics of Directed Acyclic Graphs (DAGs) in Leading Medical Journals (n = 72)

Characteristic	No./total No. (%)
Study type	
Observational	65/72 (90)
Cohort	60/65 (92)
Case-control	3/65 (5)
Cross-sectional	2/65 (3)
Interventional trial	2/72 (3)
Nonrandomized trial	1/2 (50)
Randomized clinical trial	1/2 (50)
Meta-analysis	5/72 (7)
Individual participant data meta-analysis	4/5 (80)
Study-level meta-analysis	1/5 (20)
Time point at which DAG was constructed	
Stated at design stage	26/72 (36)
Not specified	46/72 (64)
Evidence for edges	
Cited published evidence	11/72 (15)
Stated but not cited	11/72 (15)
Not specified	50/72 (70)
Expert knowledge involved	
Yes (reported)	11/72 (15)
Not specified	61/72 (85)
Variables depicted in DAGs	
Confounders	67/72 (93)
Mediators	28/72 (39)
Colliders	3/72 (4)
Inconsistency between DAG-indicated and actual adjustment strategy	39/72 (54)
Incomplete adjustment for confounders in DAGs^a^	19/67 (28)
Incomplete adjustment due to missing data	15/19 (79)
Incomplete adjustment with author’s explanation	4/19 (21)
Additional variables adjusted beyond DAG	23/72 (32)
Adjustment for variables not included in the DAG	7/23 (30)
Adjustment for other variables (not confounders)	16/23 (70)

^a^ Denominator is 67 DAGs that explicitly identified confounders.

## Discussion

The construction and use of DAGs even in leading medical journals were limited in transparency and inconsistent in practice. Fewer than half were developed at the study design stage, most did not cite evidence for edges, and nearly half showed discrepancies between DAG-indicated and actually implemented adjustment sets. However, a limitation is that we did not evaluate how these inconsistencies were associated with the studies’ effect estimates.

These gaps suggest that DAGs, while increasingly visible, are not yet being used to their full potential. While earlier work documented the availability and basic reporting of DAGs,^4,5^ our findings highlight persistent gaps in edge justification, design-stage development, and analytic concordance in top-tier clinical journals. These dimensions extend prior evaluations by demonstrating that, even in journals with the strongest methodological expectations, DAGs remain underjustified and inconsistently implemented.

Future progress will require integrating DAGs into standard frameworks for individual evidence appraisal, such as Grading of Recommendations Assessment, Development, and Evaluation, to improve reproducibility in confounding bias assessment.^6^ Clearer reporting of DAG construction and explicit justification for analytic choices will be essential to enhance credibility and strengthen the validity of the best available evidence.

## Notes

Supplement 1. eMethods. Search Strategy

Supplement 2. Data Sharing Statement
