# Supplementary material for: Construction and Application of Directed Acyclic Graphs in Leading Medical Journals
Source: JAMA Netw Open. 2026 Jan 14;9(1):e2553803. doi: 10.1001/jamanetworkopen.2025.53803 (PMC12805448; doi:10.1001/jamanetworkopen.2025.53803)
Supplement: Supplement 1. — eMethods. Search Strategy [file jamanetwopen-e2553803-s001.pdf]

Supplemental Online Content

Deng G, Du J. Construction and application of directed acyclic graphs in leading medical journals. *JAMA Netw Open*. 2026;9(1):e2553803.
doi:10.1001/jamanetworkopen.2025.53803

eMethods. Search Strategy

This supplemental material has been provided by the authors to give readers additional information about their work.

© 2025 Deng G et al. *JAMA Netw Open*.

eMethods. Search Strategy

We searched the official websites of The New England Journal of Medicine (NEJM), The Lancet, JAMA, and The BMJ for full-text articles using the following keywords: “directed acyclic graph(s)”, “causal diagram(s)”, “causal graph(s)”, “graphical model”, “graphical causal model”, “DAG(s)”, and “DAGitty”.

To minimize the risk of missing eligible studies, we additionally searched four databases—PubMed, Embase, Web of Science, and PubMed Central (PMC)—using the same set of keywords and restricted the results to articles published in the four leading medical journals. The search in PMC was included because DAG-related terms are more likely to appear in the full text, and PMC provides free access to full-text articles.

Furthermore, to capture additional relevant studies that may not have been identified through keyword searches, we conducted citation searches in PubMed for articles citing “DAGitty: a graphical tool for analyzing causal diagrams” and “Robust causal inference using directed acyclic graphs: the R package 'dagitty'”. Since DAGitty and its R package are among the most widely used tools for constructing DAGs, articles citing these works may include studies applying DAGs. We subsequently filtered these citation results to retain only articles published in the four target journals.

The search was conducted on November 10, 2025.

© 2025 Deng G et al. *JAMA Netw Open*.
