# Supplementary material for: Construction and Application of Directed Acyclic Graphs in Leading Medical Journals
Source: JAMA Netw Open. 2026 Jan 14;9(1):e2553803. doi: 10.1001/jamanetworkopen.2025.53803 (PMC12805448; doi:10.1001/jamanetworkopen.2025.53803)
Supplement: Supplement 2. — Data Sharing Statement [file jamanetwopen-e2553803-s002.pdf]

Data Sharing Statement

Deng. Construction and Application of Directed Acyclic Graphs in Leading Medical Journals. *JAMA Netw Open*. Published January 14, 2026. doi:10.1001/jamanetworkopen.2025.53803

Data
Data available: Yes
Data types: Data (not involving human participants), Data dictionary
How to access data: Jian Du, E-mail:dujian@bjmu.edu.cn
When available: With publication

Supporting Documents
Document types: None

Additional Information
Who can access the data: Researchers whose proposed use of the data has been approved
Types of analyses: for any purpose
Mechanisms of data availability: after approval
